# Plant Salinity Sensors: Current Understanding and Future Directions

**DOI:** 10.3389/fpls.2022.859224

**Published:** 2022-04-07

**Authors:** Cheng-Feng Wang, Guo-Liang Han, Zong-Ran Yang, Yu-Xia Li, Bao-Shan Wang

**Affiliations:** Shandong Provincial Key Laboratory of Plant Stress Research, College of Life Sciences, Shandong Normal University, Jinan, China

**Keywords:** salt stress, root, salt-stress sensor or receptor, signal transduction, salt tolerance

## Abstract

Salt stress is a major limiting factor for plant growth and crop yield. High salinity causes osmotic stress followed by ionic stress, both of which disturb plant growth and metabolism. Understanding how plants perceive salt stress will help efforts to improve salt tolerance and ameliorate the effect of salt stress on crop growth. Various sensors and receptors in plants recognize osmotic and ionic stresses and initiate signal transduction and adaptation responses. In the past decade, much progress has been made in identifying the sensors involved in salt stress. Here, we review current knowledge of osmotic sensors and Na^+^ sensors and their signal transduction pathways, focusing on plant roots under salt stress. Based on bioinformatic analyses, we also discuss possible structures and mechanisms of the candidate sensors. With the rapid decline of arable land, studies on salt-stress sensors and receptors in plants are critical for the future of sustainable agriculture in saline soils. These studies also broadly inform our overall understanding of stress signaling in plants.

## Introduction

Soil salinization poses a significant challenge for crop growth and yield, affecting about 1125 million hectares worldwide (Hossain, 2019). Of this land, 20% is irrigated, especially in North America, Oceania, and the Middle East. In Egypt, salinization affects up to 30% of irrigated land (Goossens et al., 1999). By 2050, half of the world’s irrigated land is expected to be salinized; the amount of arable land suitable for conventional crop farming is predicted to be reduced by approximately three hectares every minute (Shabala et al., 2015). At the same time, the global population is predicted to reach 9.6 billion by 2050 (UN-News, 2013). Elucidating the mechanisms of plant salt tolerance will be required to improve crop productivity in saline soils and meet the nutritional needs of a growing population (Munns and Tester, 2008; Liu et al., 2020). Identifying salt-stress sensors is a key step for understanding the mechanisms of salt tolerance in plants.

In plants, high salinity causes two direct stresses: ionic and osmotic, which induce secondary stresses such as oxidative stress (Zhu, 2001; Yang and Guo, 2018). Plants have evolved a variety of sensors and receptors (i.e., the components that sense stresses, hereafter referred to as sensors) that detect ionic, osmotic, and reactive oxygen species (ROS) signals to trigger downstream signal transduction pathways and prevent salt damage (Huang et al., 2012; Novaković et al., 2018; Amir et al., 2019; Mukarram et al., 2021). These stress sensors are located on the cell surface, in the endomembrane system, and in the cytoplasm (Zhu, 2016). Primary salt-stress sensors (i.e., those recognizing osmotic or ionic stress) may include pectins in the cell wall, plasma membrane lipids such as glycosyl inositol phosphorylceramide (GIPC), and plasma membrane proteins such as receptor-like kinases (RLKs), protein kinases, and ion transporters, etc. However, besides the identification of the Na^+^ receptor GIPC, little progress has been made in identifying salt-stress sensors (Yang and Guo, 2018; Jiang et al., 2019; Yu et al., 2020; Saddhe et al., 2021; Chaudhry et al., 2022), because complete knockouts of the corresponding genes are either lethal or produce no change in phenotype due to functional redundancy. Another challenge is that roots, which are the first plant organ to encounter the salinity stress, are more difficult to study than shoots. Many reviews have covered progress in elucidating downstream signaling pathways for salt and osmotic stresses (Mahajan et al., 2008; Zhu, 2016; Zhao et al., 2020; Chen et al., 2021), but identifying plant sensors and analyzing how they sense and transmit signals remains a complex and arduous process.

Some researchers have proposed that a true stress sensor should have the following characteristics: (1) it must be able to perceive stimuli inside or around the cell and have a distinct process for perception and transduction of stress signals; (2) its structural characteristics or activity must be directly altered by the perceived stress, and these changes must trigger signal transduction; and (3) its actions must lead to physiological and morphological adaptation of the plant to the stress (Vu et al., 2019; Lamers et al., 2020). In this review, we discuss the onset of salt stress perception and the role of the signal in the perception process, with a focus on the cell wall and plasma membrane sensors that receive osmotic, ionic, and ROS signals related to salt stress, based on experimental and bioinformatics data. We hypothesize that factors on the cell wall and cell membrane that have a similar structure to known sensors, like osmotic receptors, or share a certain function, in particular in the charged domain interacting with Na^+^ and/or Cl^–^, may be potential sensors. Therefore, we have analyzed the known sensors, and deduced a set of possible sensory factors in cells based on the properties and functions that the sensors should have. This analysis improves our understanding of the characteristics of salt-stress sensors on the cell surface and provides candidates for further characterization.

## Sensing of Salt Stress

### Location of Plant Salt-Stress Sensors

The root is typically the first organ to encounter the salt stress. Specific zones of the root are thought to play an important role in sensing and regulating the response to gravitropic, hydrotropic, phototropic, thigmotropic, and halotropic stimuli in plants (Gilroy, 2008; Muthert et al., 2020). After the root senses a stress signal, it differentially regulates cell growth and cell division on opposite sides of the root tip in a specific root zone, resulting in root bending. Auxin plays a key role in this process (Gilroy, 2008; Galvan-Ampudia and Testerink, 2011). Although the identity of primary salt-stress sensors and their detailed mechanisms of action are unclear, it can be inferred that they are located in a certain zone of the root meristem (Wu et al., 2015; Wu, 2018; Nakamura et al., 2021).

Sensing of salt stress signals by the root meristematic zone leads to a series of responses such as remodeling of root system architecture, halotropism, and abscisic acid (ABA) accumulation and translocation to the shoot, all of which ultimately result in adaptation to salinity (Galvan-Ampudia and Testerink, 2011; Shabala et al., 2016; Karlova et al., 2021). Similar salt sensing and signal transduction processes may also exist in shoot cells to detect and respond to salt ions such as Na^+^ that are transported to the shoot *via* the transpiration stream, but their identity and specific roles need to be further explored.

### Salt Stress Perception and Signal Transduction

The physiological and molecular processes of salt stress perception and signal transduction begin with signal perception. There are many signals involved in the salt stress response, including physicochemical osmotic signals and chemical signals such as Na^+^ and Cl^–^. In the cell wall, Na^+^ likely interacts with the negatively charged groups of cell wall components such as rhamnogalacturonan-II (RG-II), leading to changes in cell wall mechanical tension and cell turgor and the opening of stretch-activated ion channels such as calcium channels (Neill et al., 2001; O’Neill et al., 2004; Goldbach and Wimmer, 2007). The cell wall changes induced by salt stress may cause stretching of the root cell plasma membrane through the interaction of cell wall polysaccharides and certain membrane components such as FERONIA (FER), which ultimately leads to the opening of calcium channels (Feng et al., 2018). Recent research found that Na^+^ may bind to negatively charged components or to Na^+^ binding sites such as GIPC to cause a conformational change and open the Ca^2+^ channel (Jiang et al., 2019). In addition, the mechanical force or ROS generated by nicotinamide adenine dinucleotide phosphate (NADPH) oxidases in the plasma membrane may then activate components such as REDUCED HYPEROSMOLALITY-INDUCED [Ca^2+^]_i_ INCREASE1 (OSCA1) and cause Ca^2+^ to flow into the cytoplasm (Zhai et al., 2020).

**FIGURE 1:**
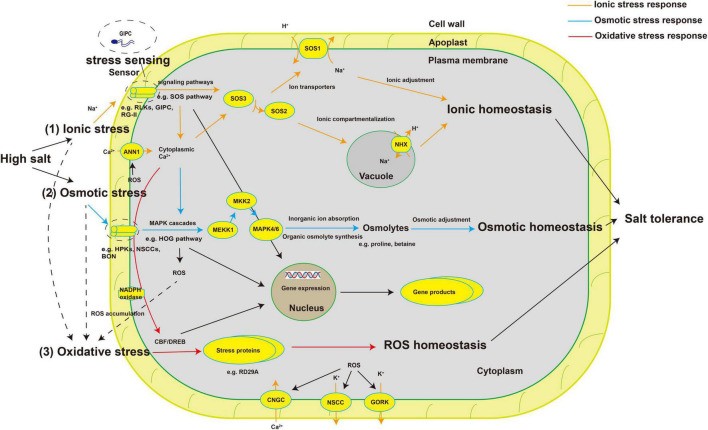
Schematic diagram of plant salt-stress sensors and signal transduction pathways involved in salt stress responses. High salt stress causes osmotic stress and ionic stress, both of which lead to the accumulation of ROS, triggering oxidative stress. (1) High concentrations of salt ions alter the concentration of ions in the cell wall, which are sensed by specific sensors or receptors (such as RLKs, GIPC, and FER), which activate signaling pathways such as the SOS pathway to redistribute ions and achieve ion homeostasis. (2) Changes in the balance between ion concentrations inside and outside the cell change the cell’s osmotic potential. The cell senses these changes through specific sensors (such as HPKs, NSCCs, and BON) and, through MAPK cascades (such as the HOG pathway), regulates the synthesis of organic osmolytes (such as proline and betaine) and the absorption of ions to achieve osmotic homeostasis. (3) The accumulation of ROS generated by plasma membrane–bound NADPH oxidase under salinity leads to secondary oxidative stress. Transcription factors such as CBF/DREB regulate the synthesis of some stress proteins (such as RD29A) to deal with the damage caused by secondary metabolites and achieve ROS homeostasis. Finally, salt tolerance is achieved through the synergy of various signaling pathways and the expression of salt tolerance–related genes. ROS, reactive oxygen species; RLKs, receptor-like protein kinases; GIPC, glycosyl inositol phosphorylceramide; FER, FERONIA; HPKs, histidine protein kinases; NSCCs, non-selective cation channels; BON, BONZAI1; SOS pathway, Salt Overly Sensitive pathway; MAPK cascades, mitogen-activated protein kinase cascades; HOG pathway, high-osmolarity glycerol pathway; NADPH, nicotinamide adenine dinucleotide phosphaten; CBF, CRT/DRE-binding factor; DREB, dehydration responsive element binding protein; ANN1, ANNEXIN1; CNGC, cyclic nucleotide-gated channel; GORK, Guard Cell Outward-Rectifying Potassium Channel; NHX, Na^+^/H^+^ exchanger.

Plants perceive stress signals and regulate the physiological and metabolic responses of cells through many signaling pathways. Common intracellular signals in stress responses include mechanical tension, Ca^2+^, ROS, cyclic nucleotides, various lipids, and plant hormones (Xiong et al., 2002; Gupta and Huang, 2014; Hou et al., 2016; Yuenyong et al., 2018). Subsequently, signal cascades including protein phosphorylation and dephosphorylation, phospholipid metabolism, cytosolic Ca^2+^ pulses, and other biochemical reactions occur under specific stresses (Agarwal et al., 2013). The well-known salt stress signal transduction pathways such as the Salt Overly Sensitive (SOS) pathway and mitogen-activated protein kinase (MAPK) cascades play an important role in the response to salt stress (Jiang et al., 2019; Chen et al., 2021). The SOS pathway regulates root ion homeostasis under salt stress (Ji et al., 2013). The high-osmolarity glycerol (HOG) MAPK cascade pathway is involved in osmotic regulation in response to hyperosmotic stress (Hohmann et al., 2007). Crosstalk also occurs between the MAPK cascades and the SOS pathway (Rodriguez et al., 2010). The SNF1-related protein kinases 2 (SnRK2) family involved in ABA signaling also plays a role in the response to salt stress. SnRK2.4 and SnRK2.10 are involved in the maintenance of root structure (McLoughlin et al., 2012). In general, salt stress signal perception may activate multiple signaling pathways, which, along with crosstalk between the pathways, enables plants to adapt to salt stress (Figure 1).

## Known and Potential Salt-Stress Sensors in Plant Cells

Excessive levels of Na^+^ and extreme osmotic stress are detected by sensors in plant cells that are as yet unidentified (Wu, 2018) and are the focus of intense research. Sensors that sense and transduce osmotic and ionic stress signals under salt stress include stretch-activated (ion) channels, transmembrane protein kinases (RLKs, histidine kinases, etc.), and cytoskeleton-associated mechanosensors (Türkan and Demiral, 2009). In the past two decades, some candidate sensors have been proposed based on the possible functional characteristics of salt-stress sensors, but their detailed mechanisms remain unclear. We predicted the three-dimensional structures of some candidate sensors using the latest authoritative protein structure prediction tool AlphaFold (Jumper et al., 2021; Supplementary Figure 1; the protein sequences are shown in Supplementary Table 1). For example, the glycine-rich protein GRP14 is a typical transmembrane protein with two extracellular regions. Both extracellular regions are negatively charged (there are 14 amino acids at the N terminus), giving this protein a physiological basis for recognizing and binding Na^+^ as a possible sensor (Supplementary Figure 2A). The predicted three-dimensional structure of GRP14 clearly shows the folding of the protein and the positions of the N and C termini (Supplementary Figure 2B). We analyzed the charge properties of amino acids 1–14 at the N terminus and found that the glutamic acid (Glu) at position 11 is a key negatively charged residue (Supplementary Figure 2C), while the other amino acids are neutral. The detail mechanisms of possible sensors like GRP14 should be investigated using point mutation of the key amino acids in the charged domain to verify that the extracellular N terminus has the ability to specifically sense Na^+^.

### Candidate Factors and Mechanisms Involved in Salt Sensing

**FIGURE 2:**
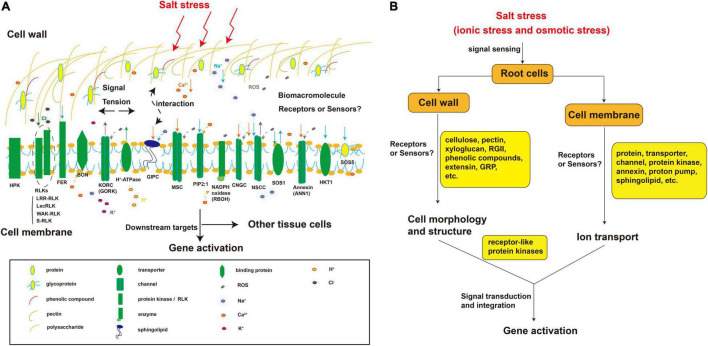
Putative components sensing physical and chemical signals in the plant root cell wall and plasma membrane under salt stress. **(A)** Cellular distribution of components known to be or that might be involved in salt stress perception; **(B)** The salt stress sensing process mediated by cell wall and plasma membrane components. The components in the cell wall and cell membrane perceive salt stress. Salt stress causes cell deformation. In the cell wall, the polysaccharides (e.g., cellulose, xyloglucan, and pectin RG-II), phenolic compounds, and proteins (e.g., GRP, glycoproteins, and extensin) undergo conformational changes due to the perception of a certain factor or binding with a certain substance such as Na^+^. Similarly, plasma membrane components such as proteins (e.g., annexin), transporters (e.g., SOS1), channels (e.g., CNGC), kinases (e.g., HPK), and sphingolipids (e.g., GIPC) also cause conformational changes in the cell wall or membrane followed by a series of biochemical reactions due to the sensing of a certain factor or binding with a certain substance such as Na^+^. These receptor-like components can also interact with each other to sense signals. RG-II, Rhamnogalacturonan II; GRP, glycine-rich protein; MSC, mechanosensory channel; CNGC, cyclic nucleotide-gated channel; HPK, histidine protein kinase; RBOH, NADPH oxidase.

Osmotic factors inhibit growth and excess ions have toxic effects on plants. Therefore, it has long been hypothesized that plant cells have mechanisms to sense osmotic and ion-specific signals (Zhu, 2003). The cell surface (cell wall or membrane) is the first part of the cell to encounter external stimuli. Presumably there are specific sensors in the cell wall or membrane for sensing changes in the concentrations of ions such as Na^+^ or Cl^–^, osmotic potential, ROS levels, and mechanical tension (Figure 2). Macromolecular cell wall and membrane components with negatively or positively charged groups may be the key players in the salt-sensing signal pathway.

Changes in cell morphology and cell wall and membrane structure under salt stress may participate in stress signaling (Zhu, 2016). Drought, osmotic stress, and salt stress cause the accumulation of ROS, cell wall stiffening, and loss of cell wall Ca^2+^ (Tenhaken, 2015; Zhu, 2016). Therefore, damage to the cell wall and changes in its composition that trigger the maintenance of cell wall integrity are thought to be a mechanism of salt sensing (Van der Does et al., 2017). Some glycine-rich proteins (GRPs) bind to RLKs in the cell wall, and phosphorylation-related proteins on the plasma membrane can combine with signal substances produced under salt stress or respond to certain stimuli, thereby causing structural changes (Ringli et al., 2001; Ryser et al., 2004; Tenhaken, 2015). Other studies have indicated that salinity induces cell wall softening, and this pectin-related cell wall defect is sensed by receptor kinases such as FER located on the plasma membrane (Feng et al., 2018). In addition, many plasma membrane components with ion binding domains such as sphingolipids (GIPC) (Jiang et al., 2019), as well as membrane-bound proteins such as cell wall integrity–related plasma membrane proteins (membrane-spanning sensors Mid2, Mtl1, and Wsc1-3 in *Saccharomyces cerevisiae*) (Jendretzki et al., 2011) and RLKs (Ye et al., 2017) are potential salt-stress sensors. Most RLKs are composed of an intracellular domain, a transmembrane (TM) domain, and an extracellular domain. The intracellular region consists of a protein kinase catalytic (PKC) domain and a juxtamembrane region. The PKC is a highly conserved domain with phosphorylation binding sites and is responsible for transducing external signals into secondary signals inside the cell (Ye et al., 2017). The juxtamembrane region separates the TM domain from the kinase region (Walker, 1994). The TM domain is composed of 22–28 amino acids and is mainly responsible for connecting the intracellular signal transmission and extracellular binding (ECLB) domains to the membrane (Ye et al., 2017). The ECLB domain is located in the extracellular region. Due to the great diversity of extracellular receptor domain structures, ECLB domains are divided into various types, which mainly function in signal recognition and binding (Greeff et al., 2012).

Many RLKs are localized to the cell wall and plasma membrane. Depending on whether the overall charge of an ECLB domain is positive or negative, it may sense Cl^–^ or Na^+^, respectively. The PKC domain of RLKs may transduce signals *via* the phosphorylation of target kinases or target proteins to initiate signal transduction under salt stress. Ion transport channels and proteins are likely to be involved in the early perception of salt stress. The salinity-induced increase in ROS levels is an early signal involved in the perception process, which can activate the outward-rectifying K^+^ channel, and can also affect the transcription of the Guard Cell Outward-Rectifying Potassium Channel (GORK) genes (Demidchik et al., 2010; Tran et al., 2013; Isayenkov and Maathuis, 2019). The outward-rectifying K^+^ channel mediates a large efflux of K^+^ and may also participate in stress perception. Ca^2+^ combined with SOS3 regulates Na^+^ homeostasis through the SOS pathway, in which SOS1 and SOS5 are likely to be intracellular and extracellular Na^+^ sensors, respectively (Shi et al., 2002; Shi et al., 2003).

Beyond the cell wall or plasma membrane, physical stress signals, in theory, can be sensed anywhere in the cell. In the endoplasmic reticulum (ER), an important effect of stress is the changes in protein folding; the ER chaperone binding immunoglobulin protein (BIP) and the INOSITOL-REQUIRING PROTEIN 1 (IRE1) protein kinase interact with unfolded proteins and are considered to be ER stress sensors (Liu and Howell, 2016; Zhu, 2016). In chloroplasts, ROS and stress signals are produced by various metabolic reactions. EXECUTER1 (EX1) and EX2 participate in the signaling pathway triggered by singlet oxygen (Wagner et al., 2004), but it is not clear whether they directly sense singlet oxygen. Mitochondria and peroxisomes, like chloroplasts, produce ROS and other metabolic signals, but it is unclear how these signals are sensed.

### Putative Receptor-Like Kinase Stress Sensors in the Root Cell Wall/Membrane

#### Putative Receptor-Like Kinase Osmosensors

Many different types of RLKs are potential sensors involved in plant salt stress responses. The Leucine Rich Repeat-RLK (LRR-RLK) superfamily is one of the largest RLK families in plants. LRR-RLKs perceive osmotic stress signals and regulate drought stress responses in plants mainly through ABA signaling pathways and/or changing the function of stomata (regulating stomatal density and closure) (Ouyang et al., 2010; Ali et al., 2020; Kim et al., 2021).

STRESS-INDUCED PROTEIN KINASE1 (OsSIK1) from rice (*Oryza sativa*) is induced by drought stress. OsSIK1 is a Mn^2+^-dependent protein kinase that autophosphorylates and phosphorylates its substrates *in vitro* (Ouyang et al., 2010). Rice *FLORAL ORGAN NUMBER1* (*FON1*) is mainly expressed in roots and is a positive regulator of drought tolerance. Overexpression of *FON1* in rice increases sensitivity to ABA, and FON1 participates in the regulation of osmotic stress by mediating the expression of ABA-responsive genes (Feng et al., 2014). In addition, LRK10L1.2 from *Arabidopsis thaliana* (Lim et al., 2015), PnLRR-RLK2 from Antarctic moss (*Pohlia nutans*) (Wang et al., 2018), SbER2 from sorghum (*Sorghum bicolor*) (Li et al., 2019), and PdERECTA from poplar (*Populus deltoides*) (Li et al., 2021) improve drought tolerance by functioning in the antioxidative systems or ABA-mediated signaling pathways. However, whether these RLKs also function in salinity-induced osmotic changes needs further study.

#### Putative Receptor-Like Kinase Ionosensors

Some RLKs have been linked to ROS bursts and the closure of plasmodesmata in plasma membrane microdomains, and directly affect the movement of Na^+^ between cells (Faulkner, 2013; Shabala et al., 2015). RLKs with (putative) carbohydrate-binding domains are found among the *Catharanthus roseus* receptor-like kinase 1-like (CrRLK1L), wall-associated kinase (WAK), S-domain, and lectin-like RLK subfamilies (Gish and Clark, 2011) and may transmit information about cell wall deformation to the cell interior *via* kinase-dependent phosphorylation of target proteins. The CrRLK1L family plasma membrane protein FER accumulates under salt stress and has a domain similar to malectin, which is considered to be able to sense pectin-related wall damage (Chen et al., 2016; Feng et al., 2018). FER and other receptor kinases with a domain similar to malectin, such as THESEUS1 (THE1) (Hématy et al., 2007) and ANXUR1 and 2 (Miyazaki et al., 2009), are all potential sensors for signal transduction from the cell wall. In addition, the LEUCINE-RICH REPEAT EXTENSINS-RAPID ALKALINIZATION FACTOR PEPTIDES-FERONIA (LRXs-RALFs-FER) module of the cell wall can transduce cell wall signals (Zhao et al., 2018).

Many different types of RLKs are potential sensors involved in plant salt stress responses. One of these types of RLKs is the LRR-RLK superfamily. For example, in *Medicago truncatula*, the LRR-RLK gene *Srlk* is rapidly induced in roots in response to salt stress but not in response to mannitol or cold temperature. Promoter-β-glucuronidase (GUS) fusion experiments showed that *Srlk* is strongly induced in root epidermal cells under salt stress. Knockdown or knockout of *Srlk* by RNA interference (RNAi) or TILLING prevented the inhibition of root growth under high salt conditions (De Lorenzo et al., 2009). In addition, *srlk* mutants accumulated significantly less Na^+^ than control plants. *Srlk* is expressed only in root tissues, and its down-regulation blocks the early responses to salt stress in roots (De Lorenzo et al., 2009). NtLRR1, a member of the LRR-RLK superfamily, was isolated from tobacco (*Nicotiana tabacum*) and is located in the cell wall. NtLRR1 is a polygalacturonase-inhibiting protein and plays a dual role in salt stress responses (Xu et al., 2009). Another LRR-RLK family member, MDIS1-INTERACTING RECEPTOR LIKE KINASE2 (MIK2), is a regulator of cell wall damage responses triggered upon inhibition of cellulose biosynthesis in Arabidopsis. The functions of MIK2 both overlap and are distinct from the functions of THE1, a malectin-like receptor kinase involved in sensing cell wall integrity. Interestingly, *mik2* mutants showed defects in the NaCl response, but not in the THE1-dependent response to osmotic stress (Van der Does et al., 2017).

Lectin receptor-like kinases (LecRLKs) can convert external stimuli into intracellular signals and play an important regulatory role in plant development and responses to environmental stress (Guo et al., 2019). LecRLKs sense stress signals and activate stress response pathways (such as ROS and SOS) to minimize the toxic effects of Na^+^ (Lin et al., 2008). The protein kinase *SALT INTOLERANCE1* (*SIT1*) in rice is mainly expressed in root epidermal cells, and its expression is rapidly induced by NaCl. SIT1 mediates salt stress signal transmission from the cell surface to the intracellular MAPK module, playing a direct role in the perception of environmental signals. Under salt stress, the protein kinase activity of SIT1 increased, and SIT1 phosphorylated its downstream effectors MAPK3 and MAPK6 (Li et al., 2014). SIT1 also promotes the accumulation of ROS under salt stress, resulting in plant growth inhibition or even death, a process dependent on MAPK3/6 and ethylene signaling (Li et al., 2014). In pea (*Pisum sativum*), LecRLKs are induced under high-salt stress and participate in the regulation of salt stress responses. Under high salt stress, together with the G protein signaling and ER stress response pathways, PsLecRLKs reduce Na^+^ and K^+^ levels by up-regulating water channels (such as common AQP, PIP1, and PIP2 aquaporin) and ion transporters [such as Na^+^/H^+^ exchangers (NHXs), high-affinity K^+^ transporter HKT1, H^+^-PPase AVP1, and SOS1] (Ye et al., 2017). The S-locus LecRLK subfamily proteins GsSRK-f and GsSRK-t from wild soybean (*Glycine soja*) (Sun et al., 2018) and the OsLecRLKs (Passricha et al., 2020) from rice have the similar functions.

Wall-associated receptor-like kinases (WAK-RLKs) contain an extracellular domain that can connect to molecules in the cell wall. This domain receives extracellular stimuli and then transmits the information through the plasma membrane to the protein’s cytoplasmic serine/threonine kinase domain to initiate downstream signaling (Kohorn and Kohorn, 2012). As a subfamily of RLKs, WAK-RLKs are characterized by the presence of extracellular epidermal growth factor-like domains (Wang et al., 2019). SlWAK1 is a kinase involved in salt stress tolerance in tomato (*Solanum lycopersicum*). *slwak1* mutants are tolerant to Na^+^ stress, but not to osmotic stress. A study found that the tolerance of these mutants to ionic stress had nothing to do with the Na^+^ transporter, but rather was due to the weak ability of their roots to transport water and solutes to the shoots (Meco et al., 2020).

S-domain receptor-like kinases (S-RLKs) are composed of three domains: the S domain, the transmembrane domain, and the kinase domain. The S domain is characterized by the sequence WQSFDXPTDTFL, called the PTDT-box, where X and F represent any non-conserved and aliphatic amino acid residues, respectively (Jose et al., 2020). The rice S-RLK *OsSIK2* is induced by NaCl treatment. *OsSRK1* expression was also induced by ABA, and *OsSRK1* overexpression lines were more sensitive to ABA than the wild type. Therefore, OsSRK1 may be involved in salt tolerance mediated by an ABA-dependent signaling pathway (Zhou et al., 2020).

**FIGURE 3:**
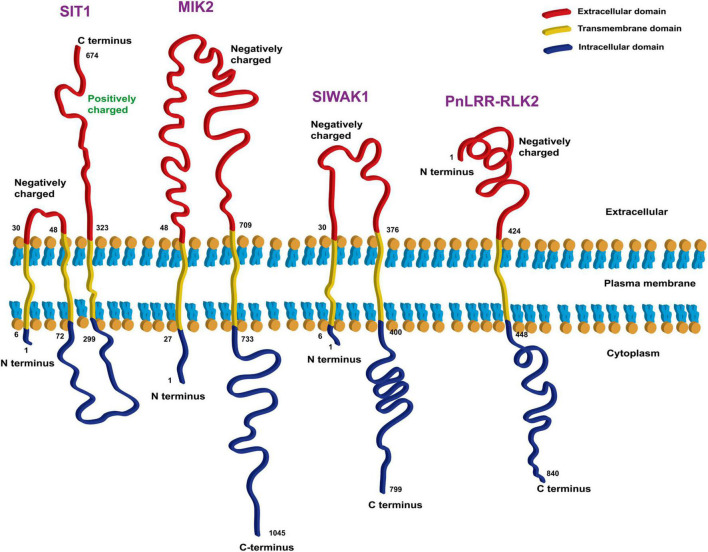
Bioinformatic analysis of transmembrane domains of four RLKs that are candidate ionic sensors. The online tools TMHMM Server v. 2.0 (http://www.cbs.dtu.dk/services/TMHMM/) and Expasy ProtParam (https://web.expasy.org/protparam/) were used for the analysis.

According to the reports discussed above, some RLKs are specifically expressed in the epidermal cells (such as Srlk) or cell walls (such as NtLRR1) of roots and specifically sense ionic stress (such as MIK2) rather than osmotic stress or other types of stress. We analyzed the extracellular domains of plasma membrane-bound RLKs and found that most of them are negatively charged, with only the second extracellular domain of SIT1 being positively charged (Figure 3). Thus, these RLKs are possible ionic sensors, sensing Na^+^ signals through negatively charged domains or Cl^–^ signals through positively charged domains. However, further studies are needed to determine whether these RLKs are involved in ionic stress signaling, and if so, their specific mechanisms need to be identified.

### Putative Sensors in the Root Cell Wall

As discussed earlier, components of the cell wall that respond to the structural changes caused by salt stress may act as sensors, but few reports have clearly shown this. Components including hydroxyproline-rich glycoproteins, WAKs, CrRLK1L proteins such as FER, and leucine-rich repeat extensins (LRXs) such as LRX_3_, LRX_4_, and LRX_5_ were identified to be involved in recognizing and relaying salt stress signals by monitoring Na^+^ and Ca^2+^ levels, and wall integrity (Li, 1990; Caffall and Mohnen, 2009; Zhao et al., 2020; Figure 2).

There is extensive cross-linking among cell wall components, which can affect the stress-induced changes in cell wall structure and signal perception. Specifically, cell wall cellulose fibrils can be cross-linked by pectins or hemicelluloses such as xyloglucan (Mohnen, 2008; Wolf et al., 2012). Pectins can be cross-linked through rhamnogalacturonan II (RG-II), and RG-II is considered to be involved in sensing cell wall integrity (Voxeur and Höfte, 2016). Pectins can also be cross-linked by extensins to positively charged scaffolds (Velasquez et al., 2011), and extensins promote the dimerization of RG-II (Chormova and Fry, 2016) and can harden the cell wall through the formation of isodityrosine cross links by ROS and peroxidases (Pereira et al., 2011).

Cross-linking may also occur between cell wall glycoproteins and phenolic compounds. The cross-linking of glycoproteins and phenolic compounds mediated by ROS and peroxidases causes local hardening of the cell wall, while expansins and xyloglucan endotransglucosylases/hydrolases play a role in loosening the cell wall (Tenhaken, 2015).

Finally, the cell wall polysaccharide network binds to associated transmembrane proteins on the cell membrane, providing another mechanism for cell wall signal sensing. For example, the previously reported cell wall–related receptors are all transmembrane proteins with extracellular domains rich in *O*-mannosylated serine/threonine residues that are embedded in the cell wall polysaccharide network (Jendretzki et al., 2011; Kock et al., 2015).

In summary, a complex polysaccharide network is formed through cross-linking of cell wall components. These components affect cell wall structure and function and may play a role in sensing salt stress. However, how these components function and what role they play under salt stress remain to be explored and verified.

### Putative Sensors in the Plasma Membrane of Root Cells

Many reports have identified cell membrane components that may act as sensors in salt stress, but most of them still need to be verified. For the purposes of this discussion, possible sensors can be divided into osmotic stress and ionic stress sensors, depending on their mechanisms.

#### Putative Sensors for Osmotic Stress

When plants are first exposed to high salt stress, they sense the osmotic stress generated by high salinity and respond quickly. Osmotic stress reduces the water potential of the cells, dehydrating them and hindering critical metabolic processes. Osmosensors in plants are also called drought sensors. Plant cell osmotic receptors sense and transduce osmotic stress signals, thereby inducing the expression of downstream genes and regulating the synthesis of osmotic protective substances to avoid cell dehydration.

Some researchers proposed that hypertonic stress is sensed *via* changes in the physical state of cell membrane lipids (Stallkamp et al., 1999). Changes in the physical state of the membrane can alter the conformation of membrane proteins, providing a mechanism to recognize stress signals. The transmembrane domains of stress sensors may be specifically adapted to sense changes in the physical state of the membrane. In addition, the stress-sensitive cell wall–related protein on the plasma membrane of yeast contains an *O*-mannosylated extracellular domain rich in serine/threonine residues and may also function as a mechanical sensor (Jendretzki et al., 2011). Osmosensors are mainly divided into three types: mechanosensory channels (MSCs)/transporters, protein kinases, and RLKs (Sayeed and Baenziger, 2009).

##### Mechanosensory Channels/Transporters

Hypertonic stress tends to change the turgor pressure of plant cells and causes dehydration and deformation. MSCs may act as mechanosensors to sense cell deformation and respond to the changes brought about by osmotic stress. MSCs comprise three main types: the MscS-like (MSL) protein family, the Mid1-complenenting activity (MCA) protein family, and the Piezo protein family (Peyronnet et al., 2014). MscS channels cause membrane depolarization when activated, quickly transporting a large number of ions (Peyronnet et al., 2014), so they are suitable sensor candidates. For example, MSL8 is considered to be a sensor of membrane tension induced by hypotonic stress in Arabidopsis (Hamilton et al., 2015). MCA channels mainly regulate Ca^2+^ permeability in response to osmotic stress or mechanical stimuli (Monshausen and Haswell, 2013). The changes in cell structure caused by activation of the MCA channels may also regulate Na^+^ permeability. Piezo channels are a type of non-selective cation channel (NSCC) (Shabala et al., 2015) and may also respond to salt stress by regulating Ca^2+^ permeability. The turgor pressure changes caused by osmotic stress regulate the opening of certain ion channels on the membrane. In Arabidopsis, OSCA1 is a high osmotic stress–gated Ca^2+^ channel protein that is also considered as an osmotic stress sensor (Yuan et al., 2014; Zhai et al., 2020). Ca^2+^-responsive phospholipid-binding BONZAI (BON) proteins play an important role in regulating all osmotic stress responses and are considered a kind of sensor in the membrane (Chen et al., 2020; Saddhe et al., 2021). In addition, the Proline Porter (ProP) in *Escherichia coli*, which responds to osmotic stress by transporting H^+^ and a variety of osmo-protective organic substances into the cell, is considered to be an osmosensor (Sayeed and Baenziger, 2009).

##### Protein Kinases

Intracellular signal transduction is often achieved through phosphorylation of signaling proteins by plasma membrane–bound protein kinases. In prokaryotic and eukaryotic cells, histidine kinases have been identified as a type of osmotic sensor (Mikami et al., 2002), including OmpF protein EnvZ and sensor kinase KdpD (in *E. coli*) (Mizuno et al., 1982; Stallkamp et al., 1999), *Dictyostelium* osmosensing kinase A (DokA, in *Dictyostelium discoideum*) (Schuster et al., 1996), histidine kinase Hik33 (in *Synechocystis* sp.) (Mikami et al., 2002), and synthetic, high osmolarity-sensitive Sho1 and Sln1 (in *S. cerevisiae*) (Ren et al., 2019). These kinases sense the change in osmotic potential caused by stress, autophosphorylate the histidine residue in their domains, and transmit the phosphate group to the asparagine residue in their domains, which in turn causes downstream signal transmission. When the non-ethylene receptor HISTIDINE KINASE 1 (HK1) from Arabidopsis was overexpressed in the yeast *sln1* mutant, it could survive normally in a high salt environment and had high osmotic stress tolerance, indicating that HK1 senses and transduces osmotic stress signals to regulate stress responses (Urao et al., 1999). Some researchers proposed that calcium-dependent protein kinases phosphorylate hypothetical aquaporins in Arabidopsis, regulating their transport activity to reduce the water permeability of the plasma membrane during osmotic stress (Johansson et al., 1998).

#### Putative Sensors for Ionic Stress

The mechanism for sensing salt stress–induced ionic stress is more complicated than that for osmotic stress. The ions sensed under salt stress are Na^+^ and Cl^–^. Theoretically, there are intra- and extracellular receptors for Na^+^ and Cl^–^ that regulate ion homeostasis (Tomar et al., 2021). GIPC sphingolipid was the first Na^+^ receptor identified, and it is required for the pulse of cytosolic Ca^2+^ induced specifically by NaCl rather than by osmotic stress (Jiang et al., 2019). Therefore, mutants of *MONOCATION-INDUCED [Ca^2+^] INCREASES 1* (*MOCA1*), which is necessary for GIPC sphingolipids biosynthesis, showed reduced growth and survival under salt stress. GIPC sphingolipids directly bind external Na^+^ and trigger the opening of Ca^2+^ channels (Jiang et al., 2019). However, little is known about the Cl^–^-sensing receptors.

##### Na^+^ Channels and Transporters

In animals, Na^+^ is sensed mainly through Na^+^-selective ion channels and transporters. Na^+^-selective ion channels have not been found in plants (Hedrich, 2012), but there are proteins with Na^+^ binding sites that may act as Na^+^ receptors. The well-known SOS1 protein is a transmembrane transporter with a long amino acid tail, which is likely involved in sensing Na^+^ (Zhu, 2003; Tomar et al., 2021). Although the role of the glutamate receptor-like transporter in salt stress perception is not clear, it is known to regulate a variety of physiological functions in abiotic stress (Cheng et al., 2018; Toyota et al., 2018), and it also has a cytoplasmic tail in its structure (Alfieri et al., 2020). Long tails or loops have been found in the structures of some transporter sensors (Snf3, Rgt2) in yeast (Özcan et al., 1998), suggesting that the presence of similar domains may be a basis for searching for unknown Na^+^ sensors in plants. Mammalian Na^+^ sensors contain a DxR/KxxH motif formed by amino acid side chains (Thomson et al., 2015). The cation-H^+^ exchangers, transporters, transcription factors, and kinases containing this motif in plants may also be able to sense Na^+^ (Maathuis, 2014). In Arabidopsis, the putative Na^+^/Ca^2+^ exchanger (NCX) protein ATNCL was found to have an intracellular Na^+^-sensing domain (Wang et al., 2012), which may sense Na^+^ and activate downstream cytoplasmic Ca^2+^ signaling to regulate intracellular metabolic activities (Shabala et al., 2015). That is, the NCX may also act as a Na^+^ sensor.

##### K^+^-Related Proton Pumps and K^+^ Channels in Tandem

Proton pumps have also been proposed to sense ionic stress. K^+^ binds to the phosphorylation domain of the plasma membrane H^+^-ATPase, inducing the dephosphorylation step of its phosphorylation reaction cycle and activating the pump (Buch-Pedersen et al., 2006). In response to salt stress, K^+^-sensing GORK channels are activated by depolarization to mediate K^+^ efflux (Shabala and Cuin, 2008). Presumably, GORK channels can also act as a type of ionic sensor in tandem with the H^+^-ATPase to activate the H^+^ pump and trigger salt-induced K^+^ signaling (Shabala et al., 2015).

##### Ca^2+^-Mediated Ion Transporters

Under ionic stress, plant cells regulate metabolism by sensing the stress signal to induce Ca^2+^ signal transduction. Various non-selective cation channels (NSCCs) in the cell membrane are activated by ROS or membrane depolarization caused by an influx of Na^+^ to allow Ca^2+^ to flow into cells (Demidchik and Maathuis, 2007). The cyclic nucleotide-gated channels (CNGCs) of cyclic nucleotide receptors contain NSCCs, which are activated by the increase in cyclic nucleotides induced by salt stress, allowing Ca^2+^ to enter the cell to participate in signal transduction (Talke et al., 2003). After the increased levels of extracellular ATP induced by salt stress are sensed by plasma membrane purine receptors, the ATP also participates in the activation of NSCCs to trigger downstream signal responses (Lew and Dearnaley, 2000; Demidchik et al., 2003; Demidchik et al., 2009). G-protein-coupled receptors are mainly responsible for transmitting extracellular signals to cells (Xue et al., 2008). After detecting exogenous salt stress signals, through early Ca^2+^ influx, G-protein-coupled receptors rapidly cause membrane depolarization and anion outflow through ion channels (Dangl et al., 1995; Gilroy and Trewavas, 2001; Lu et al., 2018). Annexins (ANNs) containing Ca^2+^ binding sites mediate Ca^2+^ signal transduction (Laohavisit and Davies, 2009), thereby participating in the cytoplasmic Ca^2+^ signal response. For example, AtANN1 increases cytoplasmic Ca^2+^ levels and maintains the stability of SOS1 in response to ROS generated by salt stress (Laohavisit et al., 2012), while AtANN4 also transiently increases Ca^2+^ and SOS2 autophosphorylation under salt stress (Ma et al., 2019). NADPH oxidases (NOX) family members are involved in the production of stress-induced Ca^2+^ signals, which can cause an increase in ROS that stabilizes SOS1 transcripts (Chung et al., 2008), and it is speculated that NADPH oxidases may act as ionic sensors in tandem with NSCCs or CNGCs (Shabala et al., 2015). Rapid Ca^2+^ transients are involved in a variety of physiological responses such as responses to osmotic and salt stress, and root responses to auxin (Felix et al., 2000; Zhu, 2003; Monshausen et al., 2011; Liang et al., 2018). The increased cytoplasmic Ca^2+^ levels induced by salt stress may be a mechanism for detecting salt stress, and an analysis based on the ability to increase cytosolic Ca^2+^ levels may be a new method to find salt-stress sensors (Liang et al., 2018).

## Conclusion and Prospects

The first plant organ to contact and sense salt in the soil is the root. Plants likely use multiple coordinated sensing mechanisms to cope with salt stress through cell surface-localized osmotic and ionic stress sensors. To recognize salinity stress, plants could in theory sense osmotic, ionic, and ROS-related signals. Once salinity stress sensors perceive such signals, they trigger signal transduction *via* target protein phosphorylation, Ca^2+^-dependent pathways, and ABA-dependent and -independent pathways to induce salt stress adaptations. Although the osmotic sensor OSCA1, the Na^+^ sensor GIPC, and FER, which can sense plant cell wall changes, are known, our current understanding of plant salinity receptors and their mechanisms is still insufficient.

Identifying more sensors or receptors and their functions remains challenging. More research is needed to screen mutants with different salt tolerance phenotypes to determine the relationships between gene expression and salt perception. Current technologies such as gene editing (e.g., CRISPR-Cas9) and omics approaches (e.g., single-cell sequencing) can be widely used to screen for salt receptor mutants and identify potential receptors.

Once the ions (such as Na^+^ or Cl^–^) are recognized, absorbed, and transported to the shoot, the aboveground cells of the plant are also exposed to the ions. It will be interesting to investigate if the shoot’s ionic sensors and signaling pathways are similar or different to those of the roots.

Furthermore, it will be interesting to explore whether other plant subcellular organelles in addition to the plasma membrane (such as chloroplasts and mitochondria) participate in the recognition and signal transduction of ionic or osmotic stress.

Halophytes may have unique sensor functions that distinguish them from non-halophytes and contribute to their greater salt tolerance. Halophytes and non-halophytes differ greatly in their morphology and molecular mechanisms of salt tolerance. When looking for ionic and osmotic stress receptors in the future, we can consider comparing the phenotypes and functions of the sensors and receptors involved in the root halotropism of halophytes and non-halophytes.

Finding the receptors that sense ionic and osmotic stresses and understanding the mechanisms of stress perception will enhance our understanding of plant salt tolerance. The application of this knowledge to crop breeding to obtain varieties with enhanced salt-stress tolerance is of great significance to the future of sustainable agriculture.

## Author Contributions

C-FW and G-LH wrote this manuscript. Z-RY and Y-XL participated in the writing and modification of this manuscript. B-SW and G-LH conceptualized the idea. All authors read and approved the final manuscript.

## Conflict of Interest

The authors declare that the research was conducted in the absence of any commercial or financial relationships that could be construed as a potential conflict of interest.

## Publisher’s Note

All claims expressed in this article are solely those of the authors and do not necessarily represent those of their affiliated organizations, or those of the publisher, the editors and the reviewers. Any product that may be evaluated in this article, or claim that may be made by its manufacturer, is not guaranteed or endorsed by the publisher.

## Supplementary Material

Supplementary Figure 1 3D structure prediction of some possible sensors and receptors by AlphaFold in *Arabidopsis thaliana*. The possible sensors and receptors: **(A)** Annexin D1 (ANN1); **(B)** Sodium/calcium exchanger NCL (NCL); **(C)** Receptor-like protein kinase FERONIA (FER); **(D)** Leucine-rich repeat receptor-like protein kinase 7 (RLK7); **(E)** REDUCED HYPEROSMOLALITY- INDUCED [Ca^2 +^]_*i*_ INCREASE1 (OSCA1); **(F)** Histidine kinase 1 (HK1); **(G)** BONZAI 1 (BON); **(H)** Glycine-rich protein (GRP14); **(I)** Sodium/hydrogen exchanger 7 (NHX7); and **(J)** Wall-associated receptor kinase 2 (WAK2).

Supplementary Figure 2 Bioinformatic analysis of GRP14 as a candidate ionic sensor by TMHMM, ProtParam, and AlphaFold. **(A)** Analysis of the extracellular, transmembrane, and intracellular domains of GRP14. **(B)** 3D domain analysis of GRP14. **(C)** Analysis of the amino acid charge properties of the extracellular domain of GRP14. The online tools TMHMM Server v. 2.0 (http://www.cbs.dtu.dk/services/TMHMM/), Expasy ProtParam (https://web.expasy.org/protparam/), and AlphaFold Protein Structure Database (https://alphafold.ebi.ac.uk/) were used for the analysis.

Supplementary Table 1 The protein sequences of some possible sensors or receptors in *Arabidopsis thaliana* analyzed by AlphaFold.

## Abbreviations

ABA: abscisic acid
ANN: annexin
BON: BONZAI
CrRLK1L: *Catharanthus roseus* receptor-like kinase 1-like
CNGC: cyclic nucleotide-gated channel
ER: endoplasmic reticulum
EX1: EXECUTER1
ECLB: extracellular binding
FER: FERONIA
FON1: FLORAL ORGAN NUMBER1
Glu: glutamic acid
GRP: glycine-rich protein
GIPC: glycosyl inositol phosphorylceramide
GORK: Guard Cell Outward-Rectifying Potassium Channel
HOG: high-osmolarity glycerol
HK1: HISTIDINE KINASE 1
IRE1: INOSITOL-REQUIRING PROTEIN 1
LecRLK: lectin receptor-like kinase
LRX: leucine-rich repeat extension
LRXs-RALFs-FER: LEUCINE-RICH REPEAT EXTENSINS-RAPID ALKALINIZATION FACTOR PEPTIDES-FERONIA
LRR-RLK: Leucine Rich Repeat-RLK
MIK2: MDIS1-INTERACTING RECEPTOR LIKE KINASE2
MSC: mechanosensory channel
MscS: mechanosensitive channels of small
MCA: Mid1-complenenting activity
MAPK: mitogen-activated protein kinase
MOCA1: MONOCATION-INDUCED [Ca^2+^] INCREASES 1
MSL: MscS-like
NHX: Na^+^/H^+^ exchanger
NCX: Na^+^/Ca^2+^ exchanger
NADPH: nicotinamide adenine dinucleotide phosphate
NSCC: non-selective cation channel
PKC: protein kinase catalytic
ROS: reactive oxygen species
RLK: receptor-like kinase
OSCA1: REDUCED HYPEROSMOLALITY-INDUCED [Ca^2+^]_i_ INCREASE1
RG-II: rhamnogalacturonan-II
RNAi: RNA interference
S-RLK: S-domain receptor-like kinase
SIT1: SALT INTOLERANCE1
SOS: Salt Overly Sensitive
SnRK2: SNF1-related protein kinases 2
OsSIK1: STRESS-INDUCED PROTEIN KINASE1
THE1: THESEUS1
TM: transmembrane
WAK: wall-associated kinase
WAK-RLK: Wall-associated receptor-like kinase.
