# Supplementary figures and images for: Plant Salinity Sensors: Current Understanding and Future Directions
Source: Front Plant Sci. 2022 Apr 7;13:859224. doi: 10.3389/fpls.2022.859224 (PMC9022007; doi:10.3389/fpls.2022.859224)

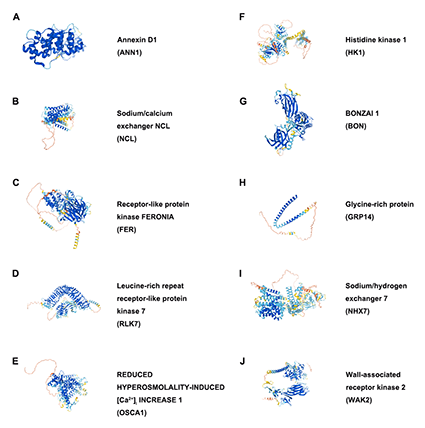

Supplement: Supplementary Figure 1 — 3D structure prediction of some possible sensors and receptors by AlphaFold in Arabidopsis thaliana. The possible sensors and receptors: (A) Annexin D1 (ANN1); (B) Sodium/calcium exchanger NCL (NCL); (C) Receptor-like protein kinase FERONIA (FER); (D) Leucine-rich repeat receptor-like protein kinase 7 (RLK7); (E) REDUCED HYPEROSMOLALITY- INDUCED [Ca2 +]i INCREASE1 (OSCA1); (F) Histidine kinase 1 (HK1); (G) BONZAI 1 (BON); (H) Glycine-rich protein (GRP14); (I) Sodium/hydrogen exchanger 7 (NHX7); and (J) Wall-associated receptor kinase 2 (WAK2). [file Image_1.TIF]

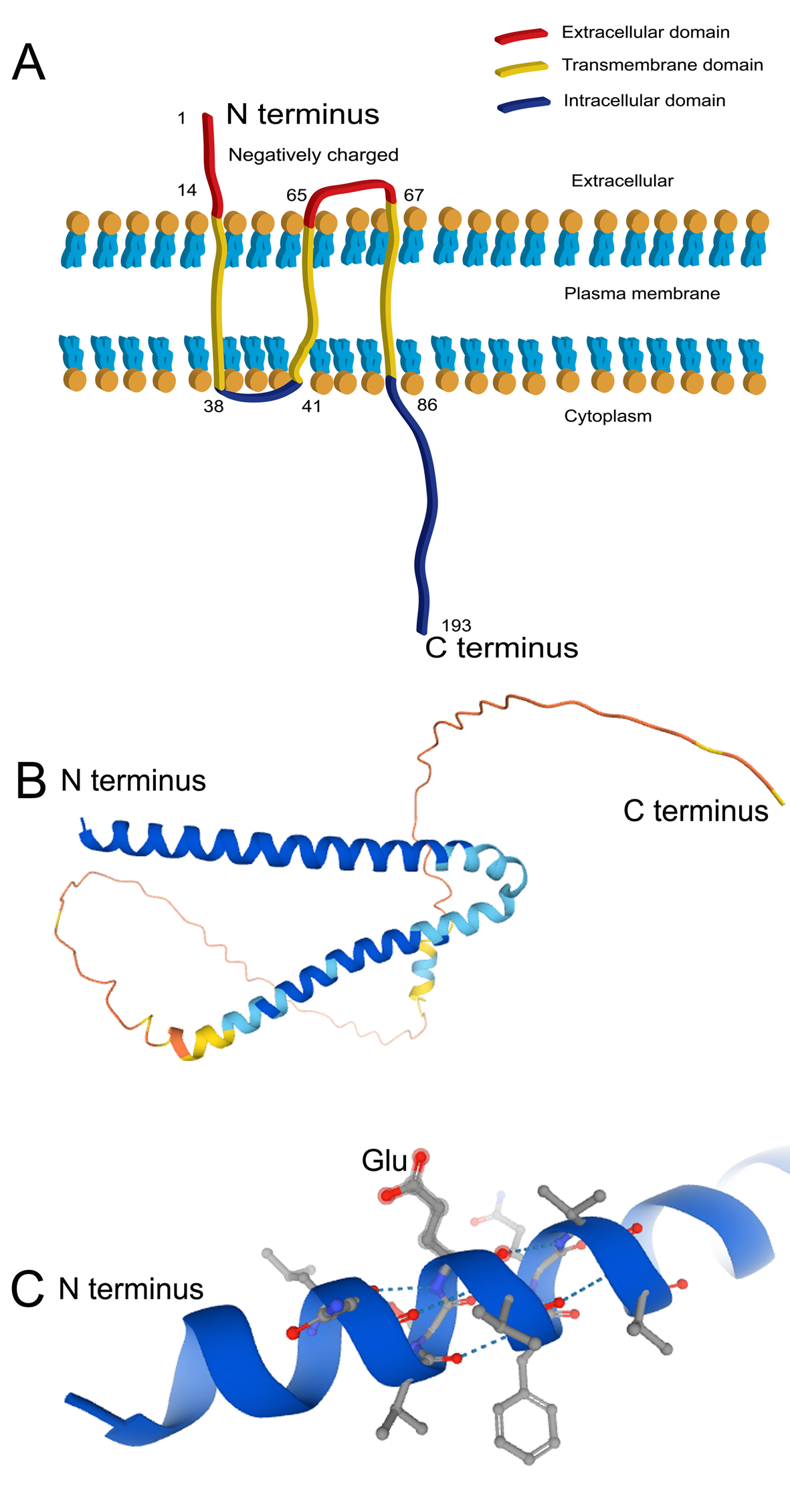

Supplement: Supplementary Figure 2 — Bioinformatic analysis of GRP14 as a candidate ionic sensor by TMHMM, ProtParam, and AlphaFold. (A) Analysis of the extracellular, transmembrane, and intracellular domains of GRP14. (B) 3D domain analysis of GRP14. (C) Analysis of the amino acid charge properties of the extracellular domain of GRP14. The online tools TMHMM Server v. 2.0 (http://www.cbs.dtu.dk/services/TMHMM/), Expasy ProtParam (https://web.expasy.org/protparam/), and AlphaFold Protein Structure Database (https://alphafold.ebi.ac.uk/) were used for the analysis. [file Image_2.TIF]
