# Supplementary material for: Plant Salinity Sensors: Current Understanding and Future Directions
Source: Front Plant Sci. 2022 Apr 7;13:859224. doi: 10.3389/fpls.2022.859224 (PMC9022007; doi:10.3389/fpls.2022.859224)
Supplement: Supplementary Table 1 — The protein sequences of some possible sensors or receptors in Arabidopsis thaliana analyzed by AlphaFold. [file Table_1.docx]

**Supplementary Table 1.** The protein sequences of some possible sensors or receptors in *Arabidopsis thaliana* analyzed by AlphaFold

| **Proteins** | **Amino acid sequence** |
| --- | --- |
| **GRP14** | **M**FSFLILQVSEVFQVVIAAVVSIVFLVLAGLTLAGSATALTITTPLFIIFSPILVPATIATAVITTGLTAG  GALGTMAAASLLSLRRRFGRRRFGGLRRFGGGRRFGGRFGKPGGGGLGGGGLPGGLGGLGGGGL  PGGLGGLGGGENPLAKISKMFGPGAAGAASGDAPPAETAPAAGAAPAAGAEPAAPPTW |
| **SIT1** | **M**RRPELIMRSLPLILFLSLGSFHLAAAAVDDQFTFDGFAGVNLTLDGTAVVTPGGLLML  TNGTTLLKGHAFYPSPLRFFHEATSGGGSSTVRSFSTAFVFGIVSEYADLSSPGLAFVVA  KSRDFSSALQSQYMGLANARNNGNASNHFLAVELDTIVNAEFGDMSDNHVGIDVDGL  ASAAADDAGYHDDRTGAFVNMSLLSRAAARVWVDFDARTSLVNVTMAPLELPKPTT  PLLSAAVNLSAVIEDEAYVGFSSSTGVVASRHYVLAWSFKMDGPAPSLNVSKLPALPV  TIARAPSNVLKILLPIASAALVSALAIAVLVIHRRRRRYAELKEEWEVAFGPHRFSYKDL  FRATNGFSDERLLGFGGFGRVYKGVLLVSRVEIAVKKVSHESRQGMKEFIAEVVSIGQ  LRHRNLVQLLGYCRQKGELLLVYDYMPNGSLDKYLYAENSKILSWAQRFRIIKGIASSI  LYLHEDWEQVVLHRDIKASNVLLDAEMNCRLGDFGLARLYDRGTDPHTTHVVGTIG  YLAPELGHTGRPSKASDIFAFGVFMLEVTCGRRPVLQDTNGGQLLLVDMVLEHWRQ  GTVTDAVDPRLQGDFAVEEASLVLKLCLLCSHPLPSARPGIRQVVQLLDGAMPLPEL  SQAHLSCNMLALMQNQMGNSCSVASSVAGNISDIPRAR |
| **MIK2** | **M**NKTNPERKISLTSFKERMACKEKPRDLQVLLIISIVLSCSFAVSATVEEANALLKWKSTFTNQTSSSKLSS  WVNPNTSSFCTSWYGVACSLGSIIRLNLTNTGIEGTFEDFPFSSLPNLTFVDLSMNRFSGTISPLWGRFSK  LEYFDLSINQLVGEIPPELGDLSNLDTLHLVENKLNGSIPSEIGRLTKVTEIAIYDNLLTGPIPSSFGNLTKLV  NLYLFINSLSGSIPSEIGNLPNLRELCLDRNNLTGKIPSSFGNLKNVTLLNMFENQLSGEIPPEIGNMTALDT  LSLHTNKLTGPIPSTLGNIKTLAVLHLYLNQLNGSIPPELGEMESMIDLEISENKLTGPVPDSFGKLTALEWL  FLRDNQLSGPIPPGIANSTELTVLQLDTNNFTGFLPDTICRGGKLENLTLDDNHFEGPVPKSLRDCKSLIRV  RFKGNSFSGDISEAFGVYPTLNFIDLSNNNFHGQLSANWEQSQKLVAFILSNNSITGAIPPEIWNMTQLSQ  LDLSSNRITGELPESISNINRISKLQLNGNRLSGKIPSGIRLLTNLEYLDLSSNRFSSEIPPTLNNLPRLYYMN  LSRNDLDQTIPEGLTKLSQLQMLDLSYNQLDGEISSQFRSLQNLERLDLSHNNLSGQIPPSFKDMLALTHVD  VSHNNLQGPIPDNAAFRNAPPDAFEGNKDLCGSVNTTQGLKPCSITSSKKSHKDRNLIIYILVPIIGAIIILSV  CAGIFICFRKRTKQIEEHTDSESGGETLSIFSFDGKVRYQEIIKATGEFDPKYLIGTGGHGKVYKAKLPNAIM  Supplementary Table 1 continues  AVKKLNETTDSSISNPSTKQEFLNEIRALTEIRHRNVVKLFGFCSHRRNTFLVYEYMERGSLRKVLENDDEA  KKLDWGKRINVVKGVAHALSYMHHDRSPAIVHRDISSGNILLGEDYEAKISDFGTAKLLKPDSSNWS  AVAGTYGYVAPELAYAMKVTEKCDVYSFGVLTLEVIKGEHPGDLVSTLSSSPPDATLSLKSISDHRLPEP  TPEIKEEVLEILKVALLCLHSDPQARPTMLSISTAFS |
| **SlWAK1** | **M**QHYQVALFSFQLPCFMLILTLATAQIIPSNTTSPPTNSTSPPTNATAPAPSPTNTITKAANITKPGCPKQCGNV  TVPYPFGIGSGCALDPMFEIDCNVTTPFIGNIQIYDISDAEMRISNFINTKCYSQTGVLIQDIPSWITLGTKSPYT  FSTLNRFIVVGCDDGAIVSGNNFANGCPSLCTSTNDIVKGKCMGFGCCQITIPKGLKFFNTTMVTTRNHSLIWS  FNPCGHSFLGEASRFEFQGIEDLSDVNFANKIRNNVPIVLDWAIGNLSCVEARKSNDYACLNNSQCVDSDTSLG  GYRCSCNSGYIGNPYIGSGCQDIDECADPNTNSCEKICTNIPGSYNCSCPEGYTGDGRKNGRGCIAPNSNSEFP  WIKFSVGMGVGFMSLVIGTTWLYFFIKKRKLIKLREKFFQQNGGLLMKQRMSSNEGGVEATKIFTAAELKKATNN  YASDRILGRGGNGIVYKGILSDNRIVAIKKSKFMDEEQVEQFINEVLILTQVNHRNVVRLFGCCLEAEVPLLVYEYI  SHGTLYEHIHNRNGAPWLSWENRLRVASETASALAYLHSSAQMPIIHRDVKSANLLLDDVYIAKVADFGASRLIP  IDQTHLATMVQGTLGYLDPEYFRTSQLTEKSDVYSFGVVLAELLTGMKPISKDRNEEEKNLAEYFVLSMRRNQLF  QILDRRVVREGSLEQLQKVAELVKSCLSLHGEDRPTMKEVASELENLRKFTKNNPWANGNGHEENEDELSDLY  TIPIESNTDIDNFSGQYSSNSYTNSSNFSGQYSSGSTSNTNSPLMKNRRAI |
| **PnLRR-RLK2** | **M**AHLKFCVAVLLLLFFSPLGTEAQNLTTLYLSSQLDALQGLWAEWNKSTPNTDSNLAGWNSTQEYPCSQGYTVF  QPNWRGVQCMLDEYNCNDTTGNRTYECNDYIIGLTLNNASITGNLPAVIGNISTLVTLELTGNPELTGPLPQEIQL  ASIYTLDLHDNAFNGILPDLPELVTILNVDLSGNEFVGEFHSQIQKWIYLQTLNLARNHFNGSIPTNAFENMTQLV  SLDLSANSLTGPLPNLAMCQNLQSLVMSNNSFNGSLPDLKMLTNLYTLKLAYNNLTGNFPLSSFVNTSVDNTLSVL  DLSGNQLTGSVTDWNGSDLGFLEELYLDNTGISGTLDITKLIAGGLVQTKLMNASGGLRSLSMMNNDITNVIYA  EGSIENITTLLRLQGNPYCQKDADDGTRCFCEQYCKNQGSMSNKKVVLIASVTSTISVLFVIAIVVAAVLYRAQRYK  RYLLLQVQQKFEEFDVKPTIFSYNELRVATRDFHVEPDMKLGEGGYGAVYKGIFPNKSMVAVKQLFVKNTKGTDD  FLNEVVLITGMKHRNLMNMKGCCLREDQRLLVYEYVDNYDIDQILLRGEHKTSLSWVVRHNICLGVARGLHYLH  ALASPRIIHRDIKASNILLDHNFEPKIADFGLALLFPDEKSHIMTMHVAGTKGYLAPEYASLGQLSEKVDVFSFGILV  LEILSGRQNIDETKPLDEVYLSKWAWKLHGEGKLMDLVDPTLSLQDNEKTEMQRLINIALLCAQNEAEQRPTMAK  VVAILQKDTESEVVVLTSGKTQPQFDTMRLLAFGNNSELTTVKEEDDEPLSSFNARSPKPASSSSSSGHDLTTKSVL  KLSEIRAR |
